# Response to Two Standardized Exercise Tests in Dogs with Different Cephalic Biotypes

**DOI:** 10.3390/vetsci12111058

**Published:** 2025-11-03

**Authors:** Brenda Reyes-Sotelo, Julio Martínez-Burnes, Ismael Hernández-Avalos, Patricia Mora-Medina, Adriana Domínguez-Oliva, Fabiola Torres-Bernal, Cynthia González-López, Daniel Mota-Rojas

**Affiliations:** 1 Doctoral Program in Biological and Health Sciences, Universidad Autónoma Metropolitana, Mexico City 04960, Mexico; 2 Facultad de Medicina Veterinaria y Zootecnia, Instituto de Ecología Aplicada, Universidad Autónoma de Tamaulipas, Ciudad Victoria 87000, Mexico; 3 Clinical Pharmacology and Veterinary Anesthesia, Biological Sciences Department, Facultad de Estudios Superiores Cuautitlán, Universidad Nacional Autónoma de México, Cuautitlán 54714, Mexico; 4 Neurophysiology, Behavior and Animal Welfare Assessment, Department of Agricultural and Animal Production, Universidad Autónoma Metropolitana, Mexico City 04960, Mexico

**Keywords:** dolichocephalic, mesocephalic, brachycephalic, brachycephalic obstructive airway syndrome

## Abstract

**Simple Summary:**

Exercise tolerance tests are used to measure cardiorespiratory performance in dogs. The cephalic index and conformational differences in dogs are related to physiological alterations within each cephalic biotype. This study aimed to evaluate the effect of moderate exercise on dolichocephalic, mesocephalic, and brachycephalic biotypes by measuring physiological and conformational parameters. The findings suggest that the 6 minute, 1000 m exercise tests and morphometric evaluation are non-invasive and can be incorporated into daily clinical evaluation. These tests are well tolerated and allow recognition of cardiorespiratory changes in dogs, including brachycephalic biotypes with obstruction grades 2 and 3.

**Abstract:**

Dogs are classified according to their total cephalic index into three biotypes: dolichocephalic, mesocephalic, and brachycephalic. The latter has emerged due to the deliberate selection of extreme phenotypic traits during breeding, which has intensified the expression of associated conformational defects and led to several medical disorders. The Brachycephalic Obstructive Airway Syndrome (BOAS) is a respiratory condition directly linked to these conformational traits. Dogs affected by BOAS present a wide range of clinical signs, including respiratory noise, exercise intolerance, syncope episodes, or even sudden death. This study aimed to evaluate craniofacial anatomical differences and similarities among dogs of different cephalic biotypes (dolichocephalic, mesocephalic, and brachycephalic) and to determine how two exercise tolerance tests—a 6 min walk and a 1000 m walk—influence physiological parameters. Eighty dogs from different breeds were included and classified according to their biotype. Morphometric data from the head, body, and limbs were obtained. Additionally, physiological parameters, including heart rate, respiratory rate, blood pressure, oxygen saturation, and rectal temperature, were evaluated before and after the tolerance tests. The results indicated that dogs tolerated both exercise tests. Dolichocephalic and mesocephalic dogs showed a greater tolerance to or greater respiratory adaptation during walking. Despite the brachycephalic biotype, a wide dispersion at a distance of 1000 m, indicating that those with a higher BOAS grade did not require emergency medical assistance during the tests. However, evidence of rostral shortening (<38 mm), together with facial foreshortening and measurements ≥ 20 mm for necks, chest circumference, and nasal fold, suggested a higher risk of airway obstruction in brachycephalic dogs diagnosed with BOAS grades 2 and 3 compared to dolichocephalic and mesocephalic dogs. This anatomical conformation was associated with significant alterations in physiological parameters including heart rate, respiratory rate, oxygen saturation below 90%, and temperature, which did not return to baseline values 10 min post-exercise. This showed significant differences between the biotypes in the distance in the 1000 m test (H = 11.74; *p* = 0.0028) and between the subdivisions (*p* = 0.0389), where G3 covered less distance than G2 (699.1 m vs. 932.77 m. These findings suggest that extreme brachycephalic conformation impairs the respiratory function and leads to thermoregulatory inefficiency, potentially compromising the animals’ survival under physical stress. Moreover, the application of safe walking tests and non-invasive morphometric measurements is suggested to facilitate prompt diagnosis of BOAS.

## 1. Introduction

The canine species exhibits significant anatomical and morphological variability, as evidenced by breed-related differences in head shape and upper airway structure. Dog breeds can be categorized by their biotype or total cephalic index (TCI), defined as the ratio of skull width to length [1]. This variability is the result of selective breeding practices to meet purebred standards, which often involve inbreeding to reinforce specific, sometimes pathological, physical traits [2,3]. These practices have resulted in phenotypic exaggerations that may result in pain, discomfort, and behavioral disorders [4]. Moreover, upper airway obstruction is associated with an elongated soft palate, stenotic nares, everted laryngeal saccules, narrowed trachea, laryngeal collapse, or paralysis [5].

Hodgman [6] documented 13 congenital abnormalities in pedigree dogs, 10 of which were associated with conformational traits, highlighting the link between phenotypic alterations and the emergence of breed-associated medical disorders. In recent years, brachycephalic breeds have gained popularity, particularly in Germany between 2002 and 2010 [7]. The UK Kennel Club also reported a 3104% increase in registrations of breeds such as French Bulldogs, 193% Pugs, and 96% Bulldogs in 2007 [8]. This is similar to reports from the Australian National Kennel Club between 1987 and 2017, which showed increases of 11.3% in French Bulldog registrations, 320% in Pug, and 324% in British Bulldog [9]. Breed standards often overlook the clinical relevance of these phenotypic abnormalities, normalizing anomalies of concern, such as head and neck malformations [10,11]. Brachycephalic breeds have been linked to rostral skeletal mutations [11], leading to Brachycephalic Obstructive Airway Syndrome (BOAS) [10,12,13,14]. BOAS severity is clinically categorized into grades 0 to 3 based on symptom intensity [15], with hallmark signs including snoring, inspiratory dyspnea, exercise intolerance, and, in severe cases, hyperthermia-induced syncope, collapse, or death [16].

Given that exercise intolerance and delayed recovery—mainly due to impaired thermoregulation—are key features of BOAS, exercise tests could serve as non-invasive, practical screening tools. The 1000 m walk test is used as a selection mechanism in brachycephalic breeding programs, and the 6 min walk test has proven effective for assessing exercise tolerance in dogs [17]. Additionally, physiological monitoring can provide relevant indicators to confirm comorbidities linked to conformational morphology [18,19,20,21,22,23]. Heat stress-related thermodynamic changes have been associated with renal, gastrointestinal, and cardiac damage [24,25], as well as inevitable respiratory compromise [10,26,27]. Therefore, this study aims to assess craniofacial anatomical differences and similarities among dogs with varying cephalic biotypes and to determine how two exercise tolerance tests: a 6 min and a 1000 m walk, influence the physiological parameters. It is hypothesized that rostral shortening is correlated with BOAS severity grades 2 and 3 in the brachycephalic phenotype, leading to more pronounced alterations in response to moderate exercise. These include increased cardiorespiratory parameters resulting from elevated inspiratory effort, transient hypoxia, and hyperthermia, which are associated with the degree of rostral shortening.

## 2. Materials and Methods

The study was conducted through a collaborative network of 10 participating veterinary clinics located in Mexico City from January to December 2024.

### 2.1. Ethical Considerations

The study was carried out exclusively with companion dogs under private ownership, whose guardians provided written informed consent prior to participation. Animal management and care were conducted in strict adherence to the Mexican Official Standard NOM-062-ZOO-1999 [28], which establishes technical specifications for the production, care, and use of experimental animals, as well as in accordance with recognized principles of applied ethology research [29]. The protocol (Approval ID: CBS.205.22) received approval from the Doctoral Program Committee in Biological and Health Sciences at the Universidad Autónoma Metropolitana, Mexico City. In addition, the study was designed and reported in alignment with the ARRIVE guidelines [30].

### 2.2. Study Population

A total of 80 dogs of various breeds were recruited. Inclusion criteria were as follows: (i) Dogs were identified as clinically healthy after a veterinary examination, with no visible signs of illness or injury. No changes were observed in the animal’s attitude, condition, physiological vital signs, hydration, or mobility, or upon head examination and oral cavity examination. No additional standardized tests were performed, such as biochemical analysis, complete blood count, urinalysis, or office diagnostic tests, including chest X-rays, ultrasound, or CT scans. (ii) Available p-to-date vaccination and deworming records. Exclusion criteria included (i) dogs younger than 1 year or older than 6 years; (ii) dogs with painful or uncomfortable conditions affecting handling; (iii) aggressive behavior; and (iv) active infectious diseases or ongoing medical treatment at the time of evaluation.

The 80 dogs were classified according to their biotype using the Total Cephalic Index (TCI), assigning them into the following groups: B1 = dolichocephalic (*n* = 20); B2 = mesocephalic (*n* = 20); and B3 = brachycephalic (*n* = 40). The brachycephalic group was further subdivided into three subgroups, according to the BOAS clinical grading: G1 = BOAS Grade 1 (*n* = 10); G2 = BOAS Grade 2 (*n* = 18); and G3 = BOAS Grade 3 (*n* = 12).

All dogs underwent a general clinical examination in which breed, age, and body weight were recorded.

### 2.3. Total Cephalic Index Evaluation

The TCI was calculated as the ratio of skull width to skull length, expressed as a percentage [31], and was used to classify dogs into B1, B2, and B3 biotypes. A trained veterinarian obtained radiographic images, assisted by a technician who positioned the dogs for left lateral and ventrodorsal skull projections. Radiographs were obtained using a TXR^®^ X-ray unit (Tingle Model 325, 125 kV, 300 mA, Vance, Al, USA) and digitized with a Fujifilm^®^ XG5000 Plus image processor via Advance Console Software Version 8.1 (Fujifilm Corporation, Tokio, Japan).

Skull width was measured from the external border of one zygomatic arch to the corresponding border on the opposite side. Maximum skull length was measured from the external occipital protuberance to the rostral end of the interincisive suture. Cephalic biotype determination thresholds were defined as follows:

Dolichocephalic (B1): TCI < 55%.

Mesocephalic (B2): TCI between 55% and 80%.

Brachycephalic (B3): TCI > 80%.

### 2.4. BOAS Functional Grading System

To further classify B3 biotype dogs, a standardized exercise test designed for evaluating BOAS was implemented. The clinical evaluation included a 3 min trot at 6–8 km/h, as per Liu et al.’s [15] protocol (see Table 1). A trained person jogged all subjects at the 10 veterinary centers without a treadmill. The Samsung Health app (Samsung Electronics Co., Ltd., version 6.30.3.011, Suwon, Republic of Korea) was used to monitor jogging speed and record the times of the functional and tolerance tests (6 min and 1000 m). Each dog received a BOAS functional grade:

Grade 0: No clinical signs, considered BOAS-free.

Grade I (Mild): Audible respiratory stertor present without exercise intolerance.

Grade II (Moderate): Significant clinical signs that require medical intervention (e.g., special management or possible surgery). The test is stopped upon the presence of these signs, even if the dogs do not finish the 3 min.

Grade III (Severe): Severe signs. Owners are advised that the dog would likely require immediate surgical correction. The test is stopped due to the presence of these signs, even if the dogs do not finish the 3 min.

This classification was performed 24 h before the four-phase exercise monitoring protocol. Based on this classification, dogs were grouped as follows:

G1 (Control): Grade 0 and Grade 1 BOAS.

G2: Grade 2 BOAS.

G3: Grade 3 BOAS.

### 2.5. Morphometric Evaluation

Thirteen conformational traits were evaluated using the measurements described by Sutter et al. [32] and Packer et al. [10]. Muzzle length (L_muzzle) was measured from the dorsal tip of the nasal plane to the stop—defined as the midpoint between the inner corners of the eyes—since the stop is often indistinct in dogs with longer muzzles and less pronounced facial angles. Cranial length (L_cranial) was measured from the stop to the external occipital protuberance, following the curvature of the skull, from between the eyes and ears to the posterior bony projection of the head.

Other measurements included eye width (W_eyes), neck length (L_neck), neck circumference (C_neck), chest circumference (C_chest), chest width (W_chest), body length (L_body), height at the withers (H_withers), and height at the tail base (H_tail). Additionally, limb circumference was measured at the right forelimb (RFL), left forelimb (LFL), right hindlimb (RHL), and left hindlimb (LHL). All measurements were taken using a flexible measuring tape, accurate to the nearest millimeter. For nasal fold thickness (Ft_nasal), a caliper was used, and measurements were recorded in millimeters.

### 2.6. Exercise Tolerance Test

All five animal groups underwent a modified exercise tolerance test adapted from Lilja-Maula et al. [33], incorporating four timepoints:

Time 1 (T1), or baseline, was recorded before the test began.

Time 2 (T2) was measured immediately after a 6 min walk (6 MWD) through a 40 m corridor, while the subject was handled on a leash by a trained person who walked all study subjects at their walking pace, without pulling. If the dogs sat down, lay down, or refused to walk, time stopped, and their test phase ended. If the animals presented severe dyspnea, cyanosis, or syncope, they were retired from the test. The test was carried out in an uncontrolled environment regarding temperature and humidity. Distance traveled was recorded in meters (m).

Time 3 (T3) was recorded immediately after completing a 1000 m walk (1000 MWD), through a 40 m corridor while handled on a leash by a trained person who walked all study subjects at their walking pace, without pulling. If the dogs sat down, lay down, or refused to walk, time stopped, and their test phase ended. If the animals presented severe dyspnea, cyanosis, or syncope, they were removed from the test. The test was carried out in an uncontrolled environment regarding temperature and humidity. Distance traveled was recorded in meters (m), and walking duration was recorded in minutes after the 6 min walk test on the same day. Time 4 (T4), or recovery, was measured 10 min after the 1000 m walk.

The exercise tolerance test started between 8:00 a.m. and 10:00 a.m. Although it was performed in non-controlled environmental conditions, the average ambient temperature in the eastern region of Mexico City was 16.75 °C, with the highest value recorded during April–August (18.6 °C). The average annual relative humidity was 61%, with the lowest levels in March at 17%, while September recorded the highest at 98%.

### 2.7. Biophysical Parameters Assessment During Mild Exercise

Biophysical parameters were measured at T1–T4 using the same veterinary multiparameter vital signs monitor (Model AM6100, VetCare^®^, Shanghai, China) at the 10 veterinary clinics. The dog was placed in sternal recumbency in a quiet environment. The skin was slightly moistened with alcohol, and the electrodes were placed in the axillary area or the proximal part of the right and left forearms. In contrast, for the left hind limb, the inguinal area or proximal internal thigh was used. For blood pressure, an appropriate cuff was used with a width covering approximately 40% of the forelimb circumference above the radius. For temperature, the probe or thermistor was placed rectally after lubrication, inserting 2–3 cm in small dogs and 4–5 cm in medium and large dogs. The parameters included heart rate (HR), respiratory rate (RR), systolic blood pressure (SBP), diastolic blood pressure (DBP), mean arterial pressure (MAP), oxygen saturation (SpO_2_), and rectal temperature (T_core). All parameters were evaluated immediately afterward. The pulsioximeter was placed on the dog’s lip.

### 2.8. Statistical Analysis

Descriptive statistics were calculated for all evaluated variables. For quantitative variables, normality was assessed using the Shapiro–Wilk test. Analysis of variance (ANOVA), followed by Tukey’s post hoc test, was used to compare morphometric variables, including L_cranial, W_eyes, L_neck, C_neck, C_chest, W_chest, L_body, H_withers, and H_tail. The same analysis was applied to limb circumferences (RFL, LFL, RHL, LHL) and E_nasal. A repeated-measures linear mixed-effects model was developed to evaluate the biophysical variables HR, RR, SBP, DBP, MAP, SpO_2_, and T_core for each of the times (T1–T4). Data were reported as mean ± standard error.

Correlations between variables and between BOAS-positive and BOAS-negative animals were analyzed using Pearson’s chi-square test.

Statistical significance was set at *p* < 0.05 for all analyses. All statistical procedures were performed using PRISM software, version 10.5.0 (GraphPad, San Diego, CA, USA).

## 3. Results

### 3.1. Breed, Age, and Weight

The most prevalent breeds in the B1 group were Dachshund (*n* = 10), Chihuahua (*n* = 3), mixed breeds (*n* = 4), American Bully (*n* = 1), Scottish Terrier (*n* = 1), and Xoloitzcuintli (*n* = 1), with an average age of 4.28 ± 0.32 years and a mean body weight of 7.36 ± 0.76 kg. In the B2 group, the breeds included Poodle (*n* = 1), American Bull Terrier (*n* = 1), Labrador Retriever (*n* = 3), Old English Sheepdog (*n* = 4), Schnauzer (*n* = 1), Beagle (*n* = 1), Chihuahua (*n* = 3), and mixed breeds (*n* = 6), with a mean age of 3.96 ± 0.34 years and a body weight of 17.23 ± 2.98 kg. For the B3 group, breeds included Pug (*n* = 23), French Bulldog (*n* = 3), English Bulldog (*n* = 4), Shih Tzu (*n* = 5), Boston Terrier (*n* = 1), and mixed breeds (*n* = 4). It can be observed that Pugs were the predominant breed, with 57.5% in B3. This difference implicates the predominant racial distribution of dogs across the 10 participating veterinary clinics in Mexico City during the sampling period.

In subgroup G1, the average age and body weight were 4.40 ± 0.50 years and 4.40 ± 0.50 kg, respectively; in G2, 2.81 ± 0.36 years and 8.74 ± 0.80 kg; and in G3, 3.59 ± 0.55 years and 10.48 ± 1.71 kg. Statistically significant differences were observed regarding the animals’ age, where B1 (4.28 ± 0.32 years), B2 (3.96 ± 0.34 years; *p* = 0.025), G1 (4.40 ± 0.50 years *p* = 0.006), and G2 (2.81 ± 0.36 years *p* < 0.0001), while G3 showed no significant differences (3.59 ± 0.55 years *p* = 0.925). B2 differed between G1 (*p* = 0.0006) and G2 (*p* = 0.0001), but not among G3 (*p* = 0.273). G1 had the oldest dogs, differing from G2 (*p* = 0.0001) (youngest dogs), while G3 fell in the middle (*p* = 0.023). G2 showed significant differences with G3 (*p* = 0.003).

Regarding weight, B1 (7.36 ± 0.76 kg) was significantly different from the other groups: B2 (17.23 ± 2.98 kg; *p* = 0.005), G1 (4.40 ± 0.50; *p* = 0.039), G2 (8.74 ± 0.80; *p* = 0.0002), and G3 (10.48 ± 1.71; *p* = 0.026). The B2 had the highest recorded body weight and was significantly different from G1 (*p* = 0.026) (animals with the lowest body weights) and G3 (*p* = 0.009). G1, containing animals with the lowest body weight, showed differences with G2 (*p* = 0.0004) and G3 (*p* = 0.014), while G2 did not differ from G3 (*p* = 0.109).

### 3.2. Morphometric Evaluation

Table 2 summarizes the morphometric values obtained for all dogs. Regarding L_muzzle, significant differences were observed between all groups: B1 differed from B2 (*p* = 0.0007), G1 (*p* = 0.006), G2 (*p* < 0.0001), and G3 (*p* < 0.0001). B2, which showed the highest value, differed significantly from G1 (*p* < 0.0001), G2 (*p* < 0.0001), and G3 (*p* < 0.0001), which had the lowest values, and particularly G2 when compared with G3 (*p* = 0.0003).

L_cranial was highest in B2, showing significant differences with B1 and G3 (*p* < 0.0001). Regarding W_eyes, B1 recorded the lowest value, with significant differences when compared to B2 (*p* = 0.0009), G1 (*p* = 0.018), G2 (*p* < 0.0001), and G3 (*p* < 0.0001).

L_neck was similar between B1, G2 (*p* = 0.393), and G3 (*p* = 0.961), with no significant differences. However, B2 obtained the highest measurement and showed significant differences compared to G1 (*p* = 0.016), G2 (*p* = 0.040), and G3 (*p* = 0.045). G1 also differed significantly from G2 and G3. C_neck was lowest in B1, which differed significantly from all other groups. The highest value was observed in G3, which differed from B1 (*p* = 0.0005) and B2 (*p* < 0.0001), but not from G1 (*p* = 0.971) or G2 (*p* = 0.354).

C_chest was lowest in B1 and showed significant differences compared to the other groups. B2 recorded the highest value and differed significantly from G1 (*p* < 0.0001) and G3 (*p* = 0.005), but not from G2 (*p* = 0.998). G1 also differed from G3 (*p* = 0.022).

### 3.3. Exercise Tolerance Test, 6 min Walk, and 1000 m

No statistically significant differences were found between the three cephalic biotypes in the 6 min test (B1 = 462 m, B2 = 451.5 m, B3 = 526.22 m; Kruskal–Wallis, H = 4.54, *p* > 0.05) or in the total time required to cover 1000 m (Kruskal–Wallis, H = 3.98, *p* > 0.05). However, the distance in the 1000 m test showed significant differences between the biotypes (Kruskal–Wallis, H = 11.74; *p* = 0.0028). In group B3 and its subdivisions (G1, G2, and G3), there were no significant differences between subdivisions for the distance covered in 6 min (G1 = 552 m, G2 = 569.83, and G3 = 439.33; *p* = 0.0585), nor for the 1000 m time (*p* = 0.8083). In contrast, statistically significant differences were observed for the total distance covered in the 1000 m test (*p* = 0.0389), where G3 covered less distance than G2 (699.1 m vs. 932.77 m), whereas G1 presented an intermediate value (876 m), without differing significantly from the other two groups. Several dogs were unable to complete the 1000 m test (G1 = 8/10; G2 = 4/18; G3 = 9/12), underscoring the need for subsequent clinical evaluations and diagnostic follow-up. A temporary loss of quadrupedal posture and cyanosis was observed in B3 but not in B1 or B2. None of the study subjects presented syncope.

### 3.4. Differences in Biophysical Parameters Among Dogs with Different Cephalic Biotypes

The results regarding differences in physiological parameters among dolichocephalic, mesocephalic, and brachycephalic biotypes are presented in Table 3. At T1, HR in group B1 was lower and significantly different from B2, G1, G2, and G3. G1 also differed from G2 and G3 (*p* = 0.045), while G2 differed from G3 (*p* = 0.0003). At T2, after the 6 min walk test, HR increased across all groups. At T3, after the 1000 m walk, group B3 showed the greatest alteration. At T4, B1 and B2 returned to baseline values, which differed significantly from those of the other groups. G1 differed from G2 (*p* < 0.0001), and G2 differed from G3 (*p* = 0.0068) 

For RR, at T1, B1 differed from G2 (*p* < 0.0001) and G3 (*p* < 0.0001). B2 differed from G1 (*p* = 0.019), G2 (*p* < 0.0001), and G3 (*p* < 0.0001). At T2, B1 differed from B2 (*p* = 0.0003) and G3 (*p* < 0.0001). B2 showed differences with G1 (*p* = 0.003), G2 (*p* = 0.005), and G3 (*p* < 0.0001). At T3, B1 differed from B2 (*p* = 0.002) and G3 (*p* < 0.0001).

MAP at T1 was lowest in B1, which was significantly lower than in all other groups. B2 only differed from G1 (*p* = 0.007). At T2, B1 was significantly lower than the rest. G2 and G3 differed (*p* = 0.0003). At T3, B1 differed from all groups. G1 differed from G2 (*p* = 0.008) and G3 (*p* < 0.0001). G2 and G3 differed (*p* = 0.0002). At T4, B1 differed from G2 (*p* < 0.0001). B2 differed only from G2 (*p* = 0.031).

SpO_2_ in B3 fell below 90% at T1, unlike in the other groups. G1 differed from G2 (*p* = 0.0001) and G3 (*p* = 0.009). At T2, B1 showed significant differences with G1 (*p* = 0.009), G2 (*p* = 0.002), and G3 (*p* = 0.009). B2 differed from G1, G2, and G3. G1 differed from G2 (*p* = 0.0008) and G3 (*p* = 0.049). At T3, G1 differed from G2 (*p* = 0.004) and G3 (*p* = 0.0003). At T4, B3 failed to recover oxygen saturation; G1 differed from G2 (*p* = 0.0002) and G3 (*p* = 0.007) (Table 3).

T_core at T1 in B1 differed from B2 (*p* < 0.0001) and G3 (*p* = 0.0004). B2 differed from G2 (*p* < 0.0001) and G3 (*p* < 0.0001). G2 differed from G3 (*p* = 0.003). At T2, B1 differed from B2, G1, G2, and G3. B2 differed significantly from G1, G2, and G3 (all *p* < 0.0001). At T3, B1 differed from B2, G2 (*p* = 0.001), and G3 (*p* < 0.0001). B2 differed from G1 (*p* = 0.052), G2, and G3 (*p* < 0.0001). G2 and G3 also differed (*p* = 0.001), showing the highest values during the 1000 m walk. At T4, B1 differed from G2 and G3. B2 differed from both (*p* < 0.0001). G1 differed from G2 (*p* = 0.009) and G3 (*p* = 0.003), while G2 differed from G3 (*p* = 0.006), with no return to baseline after 10 min.

### 3.5. Correlation Between Biophysical and Morphometric Parameters in Dogs with Different Cephalic Biotypes

Strong positive correlations (r^2^ > 0.80; *p* < 0.0001) were observed between biophysical parameters and morphometric variables in groups B1 (Table S1), B2 (Table S2), and B3 across their subgroups G1 (Table S3), G2 (Table S4), and G3 (Table S5). These correlations involved HR, RR, SBP, DBP, MAP, SpO_2_, and T_core, in association with the morphometric measurements L_muzzle, L_cranial, W_eyes, L_neck, C_neck, C_chest, W_chest, L_body, H_withers, H_tail, RFL, LFL, RHL, and LHL.

In group B3, Ft_facial also showed strong correlations with the above biophysical parameters (r^2^ > 0.80; *p* < 0.0001). Overall, a strong correlation was found across all variables in all groups. The only exceptions were T_core in groups B1 and G1, which showed moderate positive correlations (r^2^ < 0.80; *p* < 0.0001).

## 4. Discussion

This study demonstrated the application of the 6 min walk test and the 1000 m walk test in dogs with different biotypes at the clinical level. The present findings suggest that both exercise tolerance tests could be used to assess patients’ physical activity during visits to clinics and hospitals [34]. The 1000 m test was considered particularly reliable for identifying dogs affected by BOAS [35], thereby facilitating diagnosis without the use of invasive methods. It allows for additional assessment of respiratory sounds, which owners sometimes are unable to recognize as signs of disease or to normalize, as demonstrated by Packer et al. [36,37]. In this sense, Proschowsky et al. [38] found through a survey that a high percentage (79%) of French bulldog owners wanted to reacquire a dog of this breed; specifically, 36.7% would acquire one and 42.3% preferred a puppy from parents with BOAS classification, which suggests that understanding of the special needs that the breed entails should be promoted and participation increased to ensure timely diagnoses are made at a clinical level before reproduction. For this reason, from a preventive perspective, the recommendations for breeders are to establish selection programs that prioritize respiratory health over aesthetics, as has been initiated in other countries, such as Dinamarca [39] and Finland [40], where new open breed registries or crossbreeding projects have been introduced, which allow the introduction of new individuals without pedigree in the organized registry of dogs with pedigree to improve the health status and reduce the extreme features of brachycephalic dogs. These tests were generally well tolerated in all three biotypes, allowing for physiological variables to be monitored without causing syncope or heat stroke, events that could require urgent medical attention. In the present study, although both tests were performed on the same day, the animals had a recovery period between tests, which corresponded to the evaluation of physiological parameters and, additionally, to pupillometric tests, which were not considered in this study. The period between tests lasted approximately 10 to 15 min. The study encompassed a diverse range of breeds, ages, and sizes across the three biotypes, enabling the inclusion of both healthy animals and those diagnosed with BOAS at varying degrees of severity. However, B3 had a higher percentage of Pug dogs, which may limit extrapolation of these results to lead to other brachycephalic breeds. For example, Bulldogs have a wider cranial conformation and greater body mass [13], which might lead to a different cardiorespiratory response to exercise.

Although anatomical abnormalities of the upper respiratory tract in brachycephalic dogs have been studied for decades, key risk factors such as breed and the degree of brachycephaly remain relevant [41,42,43]. Our findings showed that all B3 subgroups had a TCI above 0.81, with significantly reduced muzzle and cranial lengths (*p* < 0.0001), indicating that individual craniofacial morphology within the same biotype plays a crucial role. When analyzed together with BOAS functional grading (Grades 1, 2, and 3), cranial conformation is related to BOAS pathogenesis, as described by Packer et al. [38], with skeletal-soft- tissue incongruence as a central factor. Because of this, group B3 showed a wide dispersion in the distance of the 1000 m test, which suggests heterogeneity in the physical resistance capacity, possibly linked to respiratory difficulties associated with cranial morphology and additionally to the probability of presenting subclinical signs of BOAS, as diagnosis by means of standardized tests was not performed before the functional evaluation. These tests assess the severity of the syndrome, which compromises performance and is reflected in ventilatory limitations, early fatigue, and discomfort during prolonged exercise.

Roberts et al. [44] reported a correlation between a reduced cranial length-to-width ratio and ventral tilting of the brain’s longitudinal axis (r = 0.83), as well as altered positioning of the olfactory lobe (r = 0.83), suggesting a genetically induced shift in brain positioning in this biotype. Similar findings were reported by Czeibert et al. [45] and Stone et al. [46], who developed 3D methods to highlight major cortical and subcortical brain structures, enhancing diagnostic accuracy by accounting for cranial shape variability.

Additionally, interocular width in brachycephalic dogs may affect t behavior, particularly their reduced capacity to detect horizontal movement, leading to less predatory behavior than in dolichocephalic dogs, which have forward-facing eyes and greater central visual acuity [46]. Our findings support this.

The cranial conformation of these animals—especially the splanchnocranium—is associated with increased inspiratory effort due to alterations in olfactory neuroanatomy. Air turbulence during inhalation increases negative pressure not only in the upper airways but also in the thoracic cavity. The tracheal diameter at the thoracic inlet is narrower (0.30) compared with other regions, such as the caudal cervical and intrathoracic segments (0.35 and 0.34, respectively), according to the manubrium tracheal index used in brachycephalic dogs, which may serve as a diagnostic tool for the detection of tracheal hypoplasia and as an anatomical marker of respiratory obstruction. However, the location of the tracheal luminal diameter in bulldogs may vary. Thus, these anatomical variations aggravate the airflow resistance, resulting in signs such as increased inspiratory effort, hypoxia, and reduced post-exercise recovery [47]. This reinforces our findings and their relationship with the significant alterations in oxygen saturation and rectal temperature in groups G2 and G3. This is consistent with studies conducted by Arulpagasam et al. [48], who, despite obtaining lower SpO_2_ readings in brachycephalic dogs than mesocephalic and dolichocephalic dogs, found ranges remained within the reference ranges of between 95% and 100% in the basal state. Similarly, Anyamaneecharoen et al. [49] reported values of 98 ± 1% in bulldogs free of the syndrome, 97 ± 2% in dogs with moderate BOAS, and 96 ± 1% in dogs with severe BOAS after the 6 min walk, without statistically significant differences. In this sense, the study’s relevance lies in verifying the specific modifications in brachycephalic dogs, considering that a decrease that could imply a hypoxemic process should never be considered normal or acceptable, but rather a condition that should initiate treatment. On the other hand, Ginn et al. [50] reported that nasopharyngeal turbinates were present in 82% of 53 brachycephalic dogs and 10 cats during upper airway endoscopy, with 32% of the dogs being Pugs. These anomalies increase upper airway resistance, creating a vicious cycle in which negative intraluminal pressure exacerbates soft-tissue deformities [51], leading to edema, airway collapse, and impaired thermoregulation [52,53].

Our results suggest that changes in respiratory patterns in G2 and G3 had a significant impact on thermoregulation. Although body temperature and respiratory rate increased across T1 and T2, only brachycephalic dogs exhibited signs of heat stress, likely due to inefficient airflow and respiratory conformation, consistent with findings by Davis et al. [51].

This study also confirmed that physical exercise elicited significant physiological responses in HR, RR, SBP, DBP, SpO_2_, and T_core. During the exercise tests, HR increased significantly across all biotypes, particularly in T2 and T3, reflecting increased cardiac output and oxygen demand. SpO_2_ remained stable in most groups, except G3, which showed a sustained reduction of more than 4% compared to baseline. Rovira et al. [54] observed similar HR increases (1.5× resting values) in large dog breeds, including one brachycephalic, aged 2–7 years, during active training.

Additionally, stroke volume changes were observed in B2 and G3 due to increased cardiac contractility, reduced systemic vascular resistance, and elevated central venous pressure, consistent with autonomic regulation of cardiovascular responses [55,56]. All BOAS groups displayed predominantly oral breathing at rest (G1 = 31.70 ± 4.22 rpm; G2 = 28.17 ± 1.95 rpm; G3 = 30.17 ± 2.20 rpm), but G2 and G3 exhibited more severe respiratory alterations during T2 and T3, without returning to baseline. Similarly, as observed by Gallman et al. [57], dogs with BOAS grades 1 to 3 required longer to return to baseline respiratory rate due to airflow limitation and increased inspiratory effort. The results of the present study are consistent with the observations of Lillja-Maula et al. [58], who observed that exercise testing alone might underestimate the severity of BOAS, since dogs with G2 and G3 were able to complete the tests without showing obvious clinical signs; however, combined exercise tolerance testing that is linked to the degree of respiratory signs and nasal stenosis could prove to be a reliable method to reduce the hereditary transmission of the syndrome. These findings are linked to reduced cranial and muzzle lengths (G1 = 38.00 ± 5.59 mm; G2 = 27.74 ± 3.00 mm; G3 = 28.00 ± 2.40 mm) and increased nasal fold thickness in G3 (20.14 ± 2.10 mm), which, according to Oechtering et al. [59] and Ekenstedt et al. [13], impairs nasal heat exchange, increasing thermal sensitivity.

C_neck was highest in G3 (355.10 ± 23.07 mm), differing significantly from B1 (278.60 ± 13.00 mm; *p* = 0.0005) and B2 (349.90 ± 24.03 mm; *p* < 0.0001), potentially reducing pharyngeal diameter and leading to airway collapse—an effect also linked to central obesity and metabolic syndrome in humans [60,61,62]. While additional factors, such as laryngeal collapse, age, and body condition, may influence postoperative recovery in brachycephalic dogs, even after corrective surgery, it should be taken into account, as noted by Goossens et al. [63], that physical condition cannot be fully restored. These findings are consistent with those reported by Filipas et al. [64] who indicate that an elevated body condition score may contribute to the risk of complications, including pneumonia and surgical events, and should therefore be considered within the broader set of clinical factors—particularly in dogs presenting with concurrent respiratory or gastrointestinal disease. It should be noted that the presence of subclinical or preclinical diseases could have influenced the physical performance of the biotypes, particularly B3, because examinations using other diagnostic tests (biochemistry, blood count, chest X-rays, ultrasound, etc.) before the study were not considered.

C_chest and C_width could also serve as indicators of obesity, especially in B3, since, as Gille et al. [65] noted, guardians of these breeds underestimate weight or body condition scores. This could be related to their physical and physiological performance; however, this study did not consider body condition.

In brachycephalic dogs, respiratory function was compromised, and exercise intolerance was evident, as evidenced by significant decreases in SpO_2_ from T1 to T4 in G3 (*p* = 0.0003). Fernández-Parra et al. [52] demonstrated reduced airflow (10.02–16.24 L/min) in brachycephalic dogs, compared to dolichocephalics (11.18–80.41 L/min) and mesocephalics (10.27–29.20 L/min), with increased flow resistance observed in English and French Bulldogs.

T_core increased progressively in all biotypes during exercise and decreased after a 10 min recovery period (T4). However, values remained significantly above baseline in G3, suggesting longer recovery times. A temporary loss of quadrupedal posture was observed, suggesting exercise-induced collapse, as reported in working dogs [66]. Peripheral fatigue may arise due to altered muscular action potentials, electrolyte shifts, or changes in intracellular metabolites, leading to reduced contractile force [67]. Central fatigue may indicate spinal or supraspinal disruptions associated with neurotransmitter metabolism or inhibitory afferent feedback from type III and IV muscle fibers, thereby impairing CNS-driven motor output [68].

Increased pulmonary blood flow and capillary pressure in B3 may have activated pulmonary C fibers (J receptors), triggering the somatomotor J reflex and inhibiting limb muscles. Gandevia et al. [69] noted this reflex as a key component of exercise intolerance in dogs, although it has only been studied in mesocephalic breeds, such as the Labrador Retriever. In our study, B3 limb proportions differed from B1 and B2, with only B2 and G2 showing no significant differences (*p* > 0.05), despite mesocephalic breeds generally having larger body structures than the brachycephalic dogs studied. Mach et al. [70] mention that treadmill walking tests are an alternative for diagnosing t BOAS-positive dogs in controlled environments. The syndrome may not present additional signs individually, or when the dogs are kept in uncontrolled conditions, before making the diagnosis.

### Limitations

In the present study, the non-controlled environment may have influenced individual physiological responses, especially during the hottest months; however, the aim was to assess the dogs’ real-life clinical performance. From our sample animals, only one dog had verifiable pedigree records, which may have introduced genetic variability. Moreover, as all dogs were recruited from Mexico City, the generalizability of these findings to other regions may be limited. Another important aspect is the inaccuracy of pulse oximetry measurements in conscious, restless animals, particularly in brachycephalic dogs, who do not tolerate rostral handling due to their conformation, especially those with greater shortening at the rostral level. Regarding generalizability, the predominant breed in the brachycephalic group was the young Pug. This could represent a limitation in applying the current findings to other breeds and ages thereby allowing for more precise cross-racial comparisons and strengthening the external validity of the findings. It is essential to note that the presence of subclinical or preclinical diseases may have influenced the study subjects’ physical performance, which is why we considered including preliminary diagnostic studies before exercise testing in future studies. Additionally, the lack of interest and normalization of conditions in this biotype, especially in English and French bulldogs, prevented the signing of informed consent. Therefore, future research is needed to verify breed-related differences. 

## 5. Conclusions

This study showed that both the 6-min and 1000-m walk tests were tolerated by most dogs, regardless of cephalic biotype. Thus, they are suitable for standardized clinical use, provided testing is timed appropriately and the recovery period is extended beyond 10 min.

Our findings demonstrate the negative impact of selective breeding for extreme phenotypic traits on animal welfare. Cranial and muzzle shortening (<38 mm) in brachycephalic dogs markedly increases the risk of chronic airway obstruction, as confirmed by exercise tests and correlated with BOAS severity. Neck, chest circumference, and nasal fold measurements (≥20 mm) may serve as additional indicators of airway obstruction and central obesity, potentially informing revisions to breed standards with a focus on health.

Physiological parameters indicated that dolichocephalic and mesocephalic dogs experienced fewer exercise-induced alterations compared to B3 dogs, particularly those with BOAS grades 2 and 3, who showed functional impairment and increased inspiratory effort. Extended post-exercise monitoring is recommended to assess respiratory signs, as oxygen saturation remained below 90% in all B3 groups (G1, G2, and G3), coupled with poor thermoregulatory capacity that may compromise survival in undiagnosed animals. Therefore, according to the physiological alterations observed in brachycephalic dogs, the selective breeding of dogs with extreme conformations is discouraged.

## Figures and Tables

**Table 1:** Functional classification system for Brachycephalic Obstructive Airway Syndrome based on respiratory signs before (Pre-ETT) and immediately after (Post-ETT) exercise in dogs [15].

		Respiratory Noise ^a^	Inspiratory Effort ^b^	Dyspnea/Cyanosis/Syncope ^c^
Grade 0	Pre-ETT	Not audible	Not present	Not present
	Post-ETT	Not audible	Not present	Not present
Grade I	Pre-ETT	Not audible or mild	Not present	Not present
	Post-ETT	Mild	Not present to mild	Not present
Grade II	Pre-ETT	Mild or moderate	Mild to moderate	Not present
	Post-ETT	Moderate to severe	Moderate to severe	Mild dyspnea; cyanosis or syncope not present.
Grade III	Pre-ETT	Moderate to severe	Moderate to severe	Moderate to severe dyspnea; may or may not present cyanosis. Inability to exercise.
	Post-ETT	Severe	Severe	Severe dyspnea; may or may not present cyanosis or syncope.

^a^ Respiratory noise was diagnosed by pharyngolaryngeal auscultation. Mild: only audible under auscultation; moderate: intermittent audible noise that can be heard without a stethoscope; severe: constant audible noise that can be heard without a stethoscope. ^b^ An abnormal respiratory cycle was characterized by increased effort to inhale air with the use of the diaphragm and/or inspiratory accessory muscles and/or nasal flaring with increased respiratory rate. Mild: regular breathing patterns with minimal use of the diaphragm; moderate: evident use of the diaphragm and inspiratory accessory muscles; severe: marked movement of diaphragm and accessory muscles of respiration. ^c^ Dogs with a clinical history of syncope and/or cyanosis were classified into Grade III without ETT. Mild dyspnea: signs of discomfort; moderate dyspnea: irregular breathing, signs of discomfort; severe dyspnea: irregular breathing with signs of discomfort and difficulty in breathing.

**Table 2:** Mean and standard error (SEM) of the conformational traits of dogs with distinct cephalic biotypes.

			B3			
Conformational Traits (mm)	B1 *n* = 20 Mean ± SEM	B2 *n* = 20 Mean ± SEM	G1 *n* = 20 Mean ± SEM	G2 *n* = 20 Mean ± SEM	G3 *n* = 20 Mean ± SEM	*p*-Value
Muzzle length (mm)	68.12 ± 3.15 ^a^	86.35 ± 6.44 ^b^	38.00 ± 5.59 ^c^	27.74 ± 3.00 ^d^	28.00 ± 2.40 ^e^	*p* < 0.0001
Cranial length (mm)	118.90 ± 7.42 ^a^	138.20 ± 7.41 ^b^	108.00 ± 3.87 ^b,c^	123.00 ± 5.24 ^c^	111.70 ± 6.17 ^b,c^	*p* < 0.0001
Eye width (mm)	35.50 ± 2.46 ^a^	49.60 ± 3.75 ^b^	39.95 ± 4.13 ^b^	46.09 ± 2.71 ^b^	43.42 ± 1.74 ^b^	*p* < 0.0001
Neck length (mm)	104.20 ± 6.19 ^a^	124.90 ± 7.09 ^b^	119.30 ± 8.35 ^c^	108.30 ± 6.52 ^a^	94.17 ± 7.74 ^a^	*p* < 0.0001
Neck circumference (mm)	278.60 ± 13.00 ^a^	349.90 ± 24.03 ^b^	323.20 ± 15.64 ^c^	343.70 ± 10.95 ^b,c^	355.10 ± 23.07 ^c^	*p* < 0.0001
Chest circumference (mm)	448.50 ± 15.04 ^a^	583.20 ± 47.98 ^b^	521.50 ± 24.38 ^c^	542.00 ± 19.12 ^b,c,d^	505.90 ± 30.18 ^d^	*p* < 0.0001
Chest width (mm)	185.10 ± 8.07 ^a^	263.10 ± 30.89 ^b^	179.00 ± 18.42 ^a,c^	207.10 ± 13.69 ^b,c^	219.30 ± 28.41 ^a,c^	*p* = 0.0068
Body length (mm)	446.00 ± 18.14 ^a^	599.70 ± 53.05 ^b^	520.50 ± 16.57 ^c^	546.40 ± 38.63 ^b^	541.90 ± 26.74 ^c^	*p* < 0.0001
Height at the withers (mm)	279.60 ± 12.00 ^a^	472.30 ± 39.04 ^b^	334.30 ± 14.90 ^b^	340.80 ± 10.91 ^c,d^	306.80 ± 18.87 ^d^	*p* < 0.0001
Height at the tail base (mm)	272.80 ± 14.84 ^a^	420.90 ± 42.62 ^b^	326.50 ± 14.76 ^c^	327.60 ± 14.47 ^b,c^	308.10 ± 20.35 ^b^	*p* < 0.0001
Right forelimb (mm)	124.30 ± 6.49 ^a^	166.60 ± 13.76 ^b^	134.50 ± 5.70 ^c^	142.40 ± 5.26 ^b,c^	159.80 ± 15.38 ^c^	*p* < 0.0001
Left forelimb (mm)	124.50 ± 6.46 ^a^	167.30 ± 14.05 ^b^	138.80 ± 6.81 ^c^	142.20 ± 5.43 ^b,c^	161.80 ± 14.91 ^c^	*p* < 0.0001
Right hindlimb (mm)	184.10 ± 10.78 ^a^	240.00 ± 23.04 ^b^	177.20 ± 9.05 ^c^	202.60 ± 6.85 ^b,c^	195.10 ± 13.46 ^c^	*p* < 0.0001
Left hindlimb (mm)	186.90 ± 11.68 ^a^	239.00 ± 23.09 ^b^	176.70 ± 11.67 ^c^	197.30 ± 5.96 ^b,c^	197.80 ± 12.94 ^c^	*p* = 0.0013
Nasal fold thickness (mm)			8.87 ± 1.74 ^a^	11.03 ± 1.31 ^a^	20.14 ± 2.10 ^b^	*p* = 0.0003

*n* = number of animals; SEM = Standard error mean; B1 = dolichocephalic; B2 = mesocephalic; B3 = brachycephalic; G1 = Grade BOAS 1; G2 = Grade BOAS 2; G3 = Grade BOAS 3; mm = millimeters; ^a,b,c,d,e^ = Different letters indicate significant differences (*p* < 0.05) between cephalic biotypes.

**Table 3:** Mean and standard error of the physiological parameters of dogs with distinct cephalic biotypes subjected to mild exercise tests.

			B3			
Physiological Parameters	B1 *n =* 20 Mean ± SEM	B2 *n =* 20 Mean ± SEM	G1 *n* = 10 Mean ± SEM	G2 *n* = 18 Mean ± SEM	G3 *n* = 12 Mean ± SEM	*p*-Value
Heart rate (bpm)						
T1	103.20 ± 5.83 ^a,1^	112.70 ± 5.94 ^b,1^	122.00 ± 5.46 ^c,1^	120.20 ± 5.97 ^d,1^	134.90 ± 3.50 ^e,1^	*p* < 0.0001
T2	114.90 ± 5.70 ^a,2^	113.50 ± 6.58 ^a,1^	137.40 ± 13.30 ^b,1,2,3^	131.30 ± 6.70 ^b,c,2^	142.50 ± 3.24 ^b,d,2^	*p* < 0.0001
T3	121.20 ± 6.31 ^a,2^	121.10 ± 5.52 ^a,2^	151.50 ± 7.58 ^b,2^	141.50 ± 8.64 ^c,2,3^	150.80 ± 10.06 ^b,1,2^	*p* < 0.0001
T4	115.50 ± 5.85 ^a,2^	112.70 ± 5.29 ^a,1^	136.80 ± 7.18 ^b,3^	124.30 ± 7.26 ^c,1,2,4^	140.10 ± 8.08 ^b,1,2^	*p* < 0.0001
*p*-value	*p* < 0.0001	*p* = 0.0008	*p* = 0.0002	*p* < 0.0001	*p* = 0.0018	
Respiratory rate (rpm)						
T1	24.90 ± 2.26 ^a,1^	25.60 ± 2.46 ^a,b,1^	31.70 ± 4.22 ^a,c,1^	28.17 ± 1.95 ^c,d,1^	30.17 ± 2.20 ^c,e,1^	*p* < 0.0001
T2	35.15 ± 3.65 ^a,2,3^	28.60 ± 4.45 ^b,1,2^	44.60 ± 10.63 ^a,c,1^	36.72 ± 4.83 ^a,c,1,2^	37.17 ± 2.37 ^c,2,3^	*p* < 0.0001
T3	34.40 ± 3.34 ^a,3^	28.45 ± 2.69 ^b,2^	41.60 ± 8.33 ^a,c,1^	33.78 ± 2.85 ^a,c,d,2^	43.67 ± 3.42 ^c,e,2^	*p* < 0.0001
T4	27.55 ± 2.25 ^a,4^	23.35 ± 2.21 ^b,1^	34.90 ± 7.98 ^a,c,1^	33.11 ± 3.48 ^a,c,d,1,2^	34.83 ± 2.71 ^c,e,3^	*p* < 0.0001
*p*-value	*p* < 0.0001	*p* = 0.0112	*p* > 0.05	*p* = 0.0023	*p* < 0.0001	
Systolic blood pressure (mmHg)						
T1	127.10 ± 4.43 ^a,1^	152.10 ± 6.75 ^b,1^	137.40 ± 6.84 ^c,1^	146.40 ± 7.85 ^b,c,1^	137.70 ± 6.49 ^c,1^	*p* = 0.0005
T2	133.80 ± 4.26 ^a,1^	152.40 ± 5.36 ^b,1^	148.60 ± 7.67 ^b,c,1^	143.60 ± 6.81 ^a,b,d,1^	149.10 ± 5.84 ^b,c,2^	*p* < 0.0001
T3	135.10 ± 5.60 ^a,1^	140.00 ± 6.14 ^a,c,1,2^	147.80 ± 11.62 ^a,b,c,d,1^	148.70 ± 6.49 ^a,d,1^	153.10 ± 6.82 ^b,2^	*p* = 0.0001
T4	132.60 ± 7.11 ^a,1^	141.20 ± 6.07 ^a,c,2^	139.00 ± 13.31 ^a,c,d,1^	146.40 ± 6.67 ^b,c,d,1^	145.50 ± 8.46 ^b,c,d,1,2^	*p* = 0.0045
*p*-value	*p* > 0.05	*p* = 0.0003	*p >* 0.05	*p >* 0.05	*p* = 0.0005	
Diastolic blood pressure (mmHg)						
T1	84.00 ± 4.36 ^a,1^	101.50 ± 6.83 ^b,1^	90.00 ± 7.94 ^a,b,c,1^	95.11 ± 9.73 ^a,b,c1^	97.00 ± 7.70 ^b,c1^	*p* = 0.0019
T2	82.10 ± 4.83 ^a,1^	108.10 ± 7.70 ^b,2^	101.70 ± 11.01 ^b,1^	97.00 ± 5.74 ^b,c,1^	103.40 ± 6.74 ^b,d,1^	*p* < 0.0001
T3	101.70 ± 5.96 ^a,1^	85.80 ± 5.50 ^b,1,3^	97.50 ± 7.07 ^a,b,c,1^	102.30 ± 13.02 ^c,d,1^	99.28 ± 5.01 ^c,e,1^	*p* < 0.0001
T4	89.40 ± 6.87 ^a,1^	95.80 ± 6.03 ^a,1,3^	102.20 ± 14.13 ^a,c,1^	99.22 ± 6.13 ^b,c,1^	96.08 ± 12.15 ^a,b,c,1^	*p* < 0.0001
*p*-value	*p >* 0.05	*p* < 0.0001	*p >* 0.05	*p >* 0.05	*p >* 0.05	
Mean arterial pressure (mmHg)						
T1	98.45 ± 4.03 ^a,1^	118.40 ± 6.02 ^b,1^	105.80 ± 6.81 ^c,1^	112.20 ± 8.85 ^b,c1^	110.70 ± 7.05 ^b,c1^	*p* < 0.0001
T2	99.30 ± 3.97 ^a,1^	122.80 ± 5.57 ^b,1^	117.40 ± 9.38 ^b,1^	112.50 ± 5.99 ^b,c,1^	118.70 ± 6.13 ^b,d,2^	*p* < 0.0001
T3	102.30 ± 4.93 ^a,1^	111.70 ± 5.98 ^b,1,2^	117.5 ± 12.28 ^b,1^	115.70 ± 5.11 ^c,d,1^	118.80 ± 5.48 ^c,e,1,2^	*p* < 0.0001
T4	103.80 ± 6.62 ^a,1^	111.00 ± 5.71 ^a,2^	114.40 ± 13.77 ^a,b,1^	114.9 ± 5.77 ^b,1^	112.60 ± 10.72 ^a,b,1,2^	*p* < 0.0001
*p*-value	*p* > 0.05	*p* < 0.0001	*p >* 0.05	*p >* 0.05	*p* = 0.0133	
Oxygen saturation (%)						
T1	90.40 ± 1.18 ^a,1^	94.37 ± 0.92 ^b,1^	89.20 ± 2.43 ^a,1^	83.83 ± 3.42 ^c,1^	87.17 ± 0.73 ^c,1^	*p* < 0.0001
T2	89.90 ± 1.23 ^a,1^	91.79 ± 1.64 ^a,1^	90.40 ± 1.80 ^b,1^	87.00 ± 1.65 ^c,1^	84.75 ± 1.02^,c,1,2^	*p* = 0.0002
T3	90.10 ± 2.39 ^a,1^	93.37 ± 1.14 ^b,1^	91.40 ± 1.62 ^a,b,1^	83.61 ± 2.11 ^c,1^	83.75 ± 1.18 ^c,2^	*p* = 0.0003
T4	90.40 ± 1.41 ^a,1^	93.11 ± 1.33 ^b,1^	90.60 ± 1.92 ^a,1^	85.78 ± 1.73 ^c,1^	83.58 ± 0.94 ^c,2^	*p* < 0.0001
*p*-value	*p* > 0.05	*p* > 0.05	*p* > 0.05	*p* > 0.05	*p* = 0.0003	
Rectal temperature (°C)						
T1	38.69 ± 0.08 ^a,1^	38.38 ± 0.11 ^b,1^	37.72 ± 1.01 ^a,b,c,1^	38.66 ± 0.09 ^a,c,d,1^	38.62 ± 0.07 ^c,e,1^	*p* < 0.0001
T2	38.75 ± 0.11 ^a,1,2^	38.57 ± 0.12 ^b,2^	38.93 ± 0.10 ^c,1,2^	38.96 ± 0.11 ^c,2^	38.91 ± 0.08 ^c,2^	*p* < 0.0001
T3	38.91 ± 0.09 ^a,2^	38.62 ± 0.07 ^b,2^	38.89 ± 0.16 ^a,c,1,2,3^	39.11 ± 0.10 ^c,d,3^	39.23 ± 0.07 ^c,e,2^	*p* < 0.0001
T4	38.31 ± 0.47 ^a,1,2^	38.62 ± 0.08 ^a,2^	38.57 ± 0.10 ^a,3^	38.91 ± 0.08 ^b,2^	39.12 ± 0.05 ^c,2^	*p* < 0.0001
*p*-value	*p* < 0.0001	*p* < 0.0001	*p* = 0.0005	*p* < 0.0001	*p* < 0.0001	

*n* = number of animals; SEM = standard error mean; B1 = dolichocephalic; B2 = mesocephalic; B3 = brachycephalic; G1 = Grade BOAS 1; G2 = Grade BOAS 2; G3 = Grade BOAS 3; mm = millimeters; ^a,b,c,d,e^ = different letters indicate significant differences (*p* < 0.05) between cephalic biotypes; ^1,2,3,4^ = different numerals indicate significant differences (*p* < 0.05) between evaluation times.

## Data Availability

The original contributions presented in this study are included in the article/Supplementary Materials. Further inquiries can be directed to the corresponding author(s).

## Supplementary Materials

The following supporting information can be downloaded at: https://www.mdpi.com/article/10.3390/vetsci12111058/s1, Table S1: Correlation between physiological and morphometric parameters on dogs of dolicocephalic biotype subjected to mild exercise; Table S2: Correlation between physiological and morphometric parameters on dogs of mesocephalic biotype subjected to mild exercise; Table S3: Correlation between physiological and morphometric parameters on dogs of brachycephalic biotype grade 1 BOAS subjected to mild exercise; Table S4: Correlation between physiological and morphometric parameters on dogs of brachycephalic biotype grade 2 BOAS subjected to mild exercise; Table S5: Correlation between physiological and morphometric parameters on dogs of brachycephalic biotype grade 3 BOAS subjected to mild exercise.

## Abbreviations

The following abbreviations are used in this manuscript:

Biotype	Cephalic biotype	B1 = Dolichocephalic
		B2 = Mesocephalic B3 = Brachycephalic
		G1 = Grade BOAS 1 G2 = Grade BOAS 2 G3 = Grade BOAS 3
TCI	Total Cephalic index	Percent
Pre_ETT	Pre-Exercise Tolerance Test	0 = Not present
		1 = Not audible to mild
		2 = Mild to moderate
		3 = Moderate to severe
		4 = Severe
Post_ETT	Post-Exercise Tolerance Test	0 = No present
		1 = Not audible to mild
		2 = Mild to moderate
		3 = Moderate to severe
		4 = Severe
HR	Heart rate	
RR	Respiratory rate	
SBP	Systolic blood pressure	
DBP	Diastolic blood pressure	
MAP	Mean blood pressure	
SPO2	Partial saturation of oxygen	
Tcore	Temperature	
L_muzzle	Muzzle length	
L_craneal	Craneal length	
W_eyes	Eye width	
L_neck	Neck length	
C_neck	Neck circumference	
C_chest	Chest circumference	
W_chest	Chest width	
L_Boby	Body length	
H_wither	Height at the withers	
H_Tail	Height at the tail base	
RFL	Right forelimb	
LFL	Left forelimb	
RHL	Right hindlimb	
LHL	Left hindlimb	
Ft_nasal	Nasal fold thickness	
T1	Time 1	
T2	Time 2	
T3	Time 3	
T4	Time 4	
