# Supplementary material for: Response to Two Standardized Exercise Tests in Dogs with Different Cephalic Biotypes
Source: Vet Sci. 2025 Nov 3;12(11):1058. doi: 10.3390/vetsci12111058 (PMC12656892; doi:10.3390/vetsci12111058)
Supplement: Supplementary file 1 [file vetsci-12-01058-s001.zip › vetsci-3837030-supplementary.pdf]

Supplementary material S1. Correlation between physiological and morphometric parameters on dogs of dolicocephalic biotype subjected to mild exercise tests.

	HR_B1	RR_B1	SBP_B1	DBP_B1	MAP_B1	SPO2_B1	Tcore_ B1	L_muzzle B1	L_craneal B1	W_eyes B1	L_neck B1	C_neck B1	C_chest B1	W_chest B1	L_body B1	H_wither B1	H_tail B1	RFL B1	LFL B1	RHL B1	LHL B1
HR_B1	1																				
RR_B1	0.910<br>p<0.0001	1																			
SBP_B1	0.940<br>p<0.0001	0.877<br>p<0.0001	1																		
DBP_B1	0.982<br>p<0.0001	0.952<br>p<0.0001	0.959<br>p<0.0001	1																	
MAP_B1	0.978<br>p<0.0001	0.936<br>p<0.0001	0.981<br>p<0.0001	0.995<br>p<0.0001	1																
SPO2_B1	0.864<br>p<0.0001	0.735<br>p<0.0001	0.876<br>p<0.0001	0.866<br>p<0.0001	0.876<br>p<0.0001	1															
Tcore_B1	0.635<br>p<0.0001	0.525<br>p<0.0001	0.705<br>p<0.0001	0.653<br>p<0.0001	0.675<br>p<0.0001	0.923<br>p<0.0001	1														
L_muzzle_B1	0.914<br>p<0.0001	0.921<br>p<0.0001	0.796<br>p<0.0001	0.848<br>p<0.0001	0.834<br>p<0.0001	0.602<br>p=0.005	0.436<br>p=0.055	1													
L_craneal_B1	-0.628<br>p<0.0001	´-0.628<br>p<0.0001	-0.523<br>p=0.018	-0.601<br>p<0.0001	-0.577<br>p=0.008	-0.337<br>p=0.146	-0.186<br>p=0.433	-0.638<br>p=0.002	1												
W_eyes_B1	0.501<br>p=0.003	0.539<br>p=0.003	0.363<br>p<0.0001	0.404<br>p=0.005	0.389<br>p<0.0001	0.261<br>p=0.009	0.167<br>p=0.084	0.663<br>p<0.0001	-0.310<br>p=0.003	1											
L_neck_B1	0.908<br>p<0.0001	0.897<br>p<0.0001	0.940<br>p<0.0001	0.887<br>p<0.0001	0.913<br>p<0.0001	0.938<br>p<0.0001	0.874<br>p<0.0001	0.768<br>p<0.0001	-0.444<br>p=0.500	0.478<br>p<0.0001	1										
C_neck_B1	0.850<br>p<0.0001	0.853<br>p<0.0001	0.706<br>p<0.0001	0.753<br>p<0.0001	0.743<br>p<0.0001	0.544<br>p=0.013	0.389<br>p=0.090	0.876<br>p<0.0001	-0.602<br>p=0.005	0.739<br>p<0.0001	0.776<br>p<0.0001	1									
C_chest_B1	0.915<br>p<0.0001	0.920<br>p<0.0001	0.777<br>p<0.0001	0.827<br>p<0.0001	0.814<br>p<0.0001	0.597<br>p=0.005	0.432<br>p=0.057	0.958<br>p<0.0001	-0.62<br>p=0.004	0.720<br>p<0.0001	0.788<br>p<0.0001	0.952<br>p<0.0001	1								
W_chest_B1	0.972<br>p<0.0001	0.969<br>p<0.0001	0.900<br>p<0.0001	0.928<br>p<0.0001	0.924<br>p<0.0001	0.746<br>p<0.0001	0.594<br>p=0.006	0.961<br>p<0.0001	-0.617<br>p=0.004	0.592<br>p<0.0001	0.884<br>p<0.0001	0.889<br>p<0.0001	0.945<br>p<0.0001	1							
L_body_B1	0.871<br>p<0.0001	0.871<br>p<0.0001	0.723<br>p<0.0001	0.776<br>p<0.0001	0.764<br>p<0.0001	0.553<br>p=0.011	0.389<br>p=0.090	0.905<br>p<0.0001	´-0.564<br>p=0.010	0.686<br>p<0.0001	0.760<br>p<0.0001	0.942<br>p<0.0001	0.961<br>p<0.0001	0.919<br>p<0.0001	1						
H_wither_B1	0.793<br>p<0.0001	0.811<br>p<0.0001	0.639<br>p=0.002	0.694<br>p=0.001	0.678<br>p=0.001	0.476<br>p=0.034	0.328<br>p=0.158	0.889<br>p<0.0001	-0.521<br>p=0.019	0.898<br>p<0.0001	0.706<br>p=0.001	0.938<br>p<0.0001	0.936<br>p<0.0001	0.860<br>p<0.0001	0.924<br>p<0.0001	1					
H_tail_B1	0.937<br>p<0.0001	0.939<br>p<0.0001	0.853<br>p<0.0001	0.880<br>p<0.0001	0.876<br>p<0.0001	0.718<br>p<0.0001	0.569<br>p=0.009	0.904<br>p<0.0001	-0.573<br>p=0.008	0.712<br>p<0.0001	0.878<br>p<0.0001	0.959<br>p<0.0001	0.962<br>p<0.0001	0.949<br>p<0.0001	0.940<br>p<0.0001	0.922<br>p<0.0001	1				
RFL_B1	0.860<br>p<0.0001	0.872<br>p<0.0001	0.723<br>p<0.0001	0.776<br>p<0.0001	0.762<br>p<0.0001	0.561<br>p=0.010	0.403<br>p=0.078	0.914<br>p<0.0001	-0.558<br>p=0.011	0.833<br>p<0.0001	0.768<br>p<0.0001	0.955<br>p<0.0001	0.960<br>p<0.0001	0.911<br>p<0.0001	0.955<br>p<0.0001	0.987<br>p<0.0001	0.961<br>p<0.0001	1			
LFL_B1	0.889<br>p<0.0001	0.899<br>p<0.0001	0.754<br>p<0.0001	0.807<br>p<0.0001	0.793<br>p<0.0001	0.574<br>p=0.008	0.413<br>p=0.070	0.935<br>p<0.0001	-0.61<br>p=0.004	0.776<br>p<0.0001	0.786<br>p<0.0001	0.958<br>p<0.0001	0.964<br>p<0.0001	0.938<br>p<0.0001	0.947<br>p<0.0001	0.968<br>p<0.0001	0.958<br>p<0.0001	0.987<br>p<0.0001	1		
RHL _B1	0.919<br>p<0.0001	0.920<br>p<0.0001	0.828<br>p<0.0001	0.837<br>p<0.0001	0.841<br>p<0.0001	0.727<br>p<0.0001	0.599<br>p=0.005	0.902<br>p<0.0001	-0.519<br>p=0.019	0.670<br>p<0.0001	0.893<br>p<0.0001	0.930<br>p<0.0001	0.948<br>p<0.0001	0.946<br>p<0.0001	0.964<br>p<0.0001	0.902<br>p<0.0001	0.965<br>p<0.0001	0.942<br>p<0.0001	0.937<br>p<0.0001	1	
LHLI _B1	0.889<br>p<0.0001	0.891<br>p<0.0001	0.793<br>p<0.0001	0.810<br>p<0.0001	0.81<br>p<0.0001	0.691<br>p=0.001	0.571<br>p=0.009	0.929<br>p<0.0001	´-0.512<br>p=0.021	0.751<br>p<0.0001	0.861<br>p<0.0001	0.899<br>p<0.0001	0.937<br>p<0.0001	0.940<br>p<0.0001	0.933<br>p<0.0001	0.927<br>p<0.0001	0.932<br>p<0.0001	0.943<br>p<0.0001	0.934<br>p<0.0001	0.966<br>p<0.0001	1

Supplementary material S2. Correlation between physiological and morphometric parameters on dogs of mesocephalic biotype subjected to mild exercise tests.

	HR_B2	RR_B2	SBP_B2	DBP_B2	MAP_B2	SPO2_B2	Tcore_B2	L_muzzle B2	L_craneal_ B2	W_eyes B2	L_neck B2	C_neck B2	C_chest B2	W_chest B2	L_body B2	H_wither B2	H_tail B2	RFL B2	LFL B2	RHL B2	LHL B2
HR_B2	1																				
RR_B2	0.912 p<0.0001	1																			
SBP_B2	0.970 p<0.0001	0.941 p<0.0001	1																		
DBP_B2	0.980 p<0.0001	0.915 p<0.0001	0.982 p<0.0001	1																	
MAP_B2	0.981 p<0.0001	0.927 p<0.0001	0.991 p<0.0001	0.998 p<0.0001	1																
SPO2_B2	0.888 p<0.0001	0.839 p<0.0001	0.937 p<0.0001	0.940 p<0.0001	0.943 p<0.0001	1															
Tcore_B2	0.980 p<0.0001	0.933 p<0.0001	0.987 p<0.0001	0.988 p<0.0001	0.992 p<0.0001	0.931 p<0.0001	1														
L_muzzle_B2	0.967 p<0.0001	0.960 p<0.0001	0.982 p<0.0001	0.934 p<0.0001	0.959 p<0.0001	0.924 p<0.0001	0.889 p<0.0001	1													
L_craneal_B2	0.943 p<0.0001	0.935 p<0.0001	0.944 p<0.0001	0.878 p<0.0001	0.910 p<0.0001	0.877 p<0.0001	0.856 p<0.0001	0.933 p<0.0001	1												
W_eyes_B2	0.879 p<0.0001	0.898 p<0.0001	0.883 p<0.0001	0.837 p<0.0001	0.863 p<0.0001	0.808 p<0.0001	0.801 p<0.0001	0.855 p<0.0001	0.969 p<0.0001	1											
L_neck_B2	0.871 p<0.0001	0.880 p<0.0001	0.876 p<0.0001	0.833 p<0.0001	0.859 p<0.0001	0.781 p<0.0001	0.835 p<0.0001	0.857 p<0.0001	0.969 p<0.0001	0.976 p<0.0001	1										
C_neck_B2	0.939 p<0.0001	0.960 p<0.0001	0.967 p<0.0001	0.898 p<0.0001	0.930 p<0.0001	0.881 p<0.0001	0.847 p<0.0001	0.985 p<0.0001	0.930 p<0.0001	0.852 p<0.0001	0.850 p<0.0001	1									
C_chest_B2	0.933 p<0.0001	0.939 p<0.0001	0.960 p<0.0001	0.897 p<0.0001	0.927 p<0.0001	0.868 p<0.0001	0.868 p<0.0001	0.971 p<0.0001	0.921 p<0.0001	0.831 p<0.0001	0.846 p<0.0001	0.987 p<0.0001	1								
W_chest_B2	0.885 p<0.0001	0.949 p<0.0001	0.915 p<0.0001	0.821 p<0.0001	0.861 p<0.0001	0.795 p<0.0001	0.777 p<0.0001	0.927 p<0.0001	0.924 p<0.0001	0.871 p<0.0001	0.875 p<0.0001	0.948 p<0.0001	0.928 p<0.0001	1							
L_body_B2	0.816 p<0.0001	0.946 p<0.0001	0.861 p<0.0001	0.744 p<0.0001	0.790 p<0.0001	0.692 p<0.0001	0.715 p<0.0001	0.877 p<0.0001	0.882 p<0.0001	0.858 p<0.0001	0.855 p<0.0001	0.908 p<0.0001	0.888 p<0.0001	0.959 p<0.0001	1						
H_wither_B2	0.915 p<0.0001	0.964 p<0.0001	0.943 p<0.0001	0.848 p<0.0001	0.889 p<0.0001	0.823 p=0.000	0.827 p<0.0001	0.952 p<0.0001	0.945 p<0.0001	0.880 p<0.0001	0.892 p<0.0001	0.977 p<0.0001	0.976 p<0.0001	0.973 p<0.0001	0.955 p<0.0001	1					
H_tail_B2	0.909 p<0.0001	0.958 p<0.0001	0.945 p<0.0001	0.876 p<0.0001	0.908 p=0.000	0.818 p=0.000	0.846 p<0.0001	0.946 p<0.0001	0.942 p<0.0001	0.893 p<0.0001	0.911 p<0.0001	0.961 p<0.0001	0.957 p<0.0001	0.979 p<0.0001	0.951 p<0.0001	0.984 p<0.0001	1				
RFL_B2	0.948 p<0.0001	0.980 p<0.0001	0.970 p<0.0001	0.896 p<0.0001	0.930 p=0.000	0.874 p=0.000	0.861 p<0.0001	0.976 p<0.0001	0.947 p<0.0001	0.886 p<0.0001	0.885 p<0.0001	0.984 p<0.0001	0.976 p<0.0001	0.974 p<0.0001	0.945 p<0.0001	0.990 p<0.0001	0.985 p<0.0001	1			
LFL_B2	0.947 p<0.0001	0.976 p<0.0001	0.973 p<0.0001	0.900 p<0.0001	0.933 p=0.000	0.880 p=0.000	0.855 p<0.0001	0.980 p<0.0001	0.943 p<0.0001	0.878 p<0.0001	0.874 p<0.0001	0.990 p<0.0001	0.982 p<0.0001	0.965 p<0.0001	0.936 p<0.0001	0.989 p<0.0001	0.978 p<0.0001	0.998 p<0.0001	1		
RHL_B2	0.932 p<0.0001	0.958 p<0.0001	0.965 p<0.0001	0.895 p<0.0001	0.927 p=0.000	0.870 p=0.000	0.847 p<0.0001	0.982 p<0.0001	0.919 p<0.0001	0.842 p<0.0001	0.842 p<0.0001	0.998 p<0.0001	0.988 p<0.0001	0.940 p<0.0001	0.905 p<0.0001	0.972 p<0.0001	0.956 p<0.0001	0.979 p<0.0001	0.986 p<0.0001	1	
LHL _B2	0.933 p<0.0001	0.953 p<0.0001	0.965 p<0.0001	0.900 p<0.0001	0.930 p=0.000	0.878 p=0.000	0.840 p<0.0001	0.983 p<0.0001	0.917 p<0.0001	0.837 p<0.0001	0.834 p<0.0001	0.997 p<0.0001	0.988 p<0.0001	0.936 p<0.0001	0.895 p<0.0001	0.969 p<0.0001	0.950 p<0.0001	0.977 p<0.0001	0.985 p<0.0001	0.998 p<0.0001	1

Supplementary material S3. Correlation between physiological and morphometric parameters on dogs of brachycephalic biotype grade 1 BOAS subjected to mild exercise tests.

	HR_G1	RR_G1	SBP_G1	DBP_G1	MAP_G1	SPO2_G1	Tcore_G1	L_muzzle G1	L_craneal G1	W_eyes G1	L_neck G1	C_neck G1	C_chest G1	W_chest G1	L_Body G1	H_wither G1	H_tail G1	RFL G1	LFL G1	RHL G1	LHL G1	E_Facial G1
HR_G1	1																					
RR_G1	0.965<br>p<0.0001	1																				
SBP_G1	0.940<br>p<0.0001	0.908<br>p<0.0001	1																			
DBP_G1	0.992<br>p<0.0001	0.966<br>p<0.0001	0.963<br>p<0.0001	1																		
MAP_G1	0.984<br>p<0.0001	0.953<br>p<0.0001	0.981<br>p<0.0001	0.991<br>p<0.0001	1																	
SPO2_G1	0.887<br>p<0.0001	0.795<br>p<0.0001	0.886<br>p<0.0001	0.892<br>p<0.0001	0.885<br>p<0.0001	1																
Tcore_G1	0.533<br>p<0.0001	0.387<br>p=0.014	0.520<br>p=0.001	0.511<br>p=0.001	0.523<br>p=0.001	0.705<br>p<0.0001	1															
L_muzzle_G1	0.770<br>p=0.009	0.890<br>p=0.001	0.938<br>p<0.0001	0.813<br>p=0.004	0.913<br>p<0.0001	0.690<br>p=0.027	0.396<br>p=0.258	1														
L_craneal_G1	0.813<br>p=0.004	0.910<br>p<0.0001	0.936<br>p<0.0001	0.840<br>p=0.002	0.926<br>p<0.0001	0.705<br>p=0.023	0.465<br>p=0.176	0.987<br>p<0.0001	1													
W_eyes_G1	0.979<br>p<0.0001	0.965<br>p<0.0001	0.861<br>p=0.001	0.981<br>p<0.0001	0.964<br>p<0.0001	0.928<br>p<0.0001	0.819<br>p=0.004	0.824<br>p=0.003	0.856<br>p=0.002	1												
L_neck_G1	0.851<br>p=0.002	0.958<br>p<0.0001	0.931<br>p<0.0001	0.895<br>p<0.0001	0.945<br>p<0.0001	0.853<br>p=0.002	0.561<br>p=0.091	0.929<br>p<0.0001	0.937<br>p<0.0001	0.903<br>p<0.0001	1											
C_neck_G1	0.836<br>p=0.003	0.960<br>p<0.0001	0.931<br>p<0.0001	0.892<br>p=0.001	0.937<br>p<0.0001	0.892<br>p=0.001	0.562<br>p=0.091	0.906<br>p<0.0001	0.902<br>p<0.0001	0.897<br>p<0.0001	0.987<br>p<0.0001	1										
C_chest_G1	0.857<br>p=0.002	0.959<br>p<0.0001	0.897<br>p<0.0001	0.907<br>p<0.0001	0.919<br>p<0.0001	0.942<br>p<0.0001	0.639<br>p=0.047	0.830<br>p=0.003	0.832<br>p=0.003	0.907<br>p<0.0001	0.959<br>p<0.0001	0.984<br>p<0.0001	1									
W_chest_G1	0.864<br>p=0.001	0.946<br>p<0.0001	0.927<br>p<0.0001	0.905<br>p<0.0001	0.956<br>p<0.0001	0.852<br>p=0.002	0.574<br>p=0.083	0.953<br>p<0.0001	0.945<br>p<0.0001	0.916<br>p<0.0001	0.981<br>p<0.0001	0.973<br>p<0.0001	0.934<br>p<0.0001	1								
L_Body_G1	0.949<br>p<0.0001	0.962<br>p<0.0001	0.908<br>p<0.0001	0.953<br>p<0.0001	0.968<br>p<0.0001	0.860<br>p=0.001	0.702<br>p=0.024	0.907<br>p<0.0001	0.939<br>p<0.0001	0.954<br>p<0.0001	0.953<br>p<0.0001	0.922<br>p<0.0001	0.902<br>p<0.0001	0.951<br>p<0.0001	1							
H_wither_G1	0.930<br>p<0.0001	0.982<br>p<0.0001	0.895<br>p<0.0001	0.950<br>p<0.0001	0.971<br>p<0.0001	0.908<br>p<0.0001	0.699<br>p=0.025	0.865<br>p=0.001	0.893<br>p<0.0001	0.960<br>p<0.0001	0.965<br>p<0.0001	0.951<br>p<0.0001	0.948<br>p<0.0001	0.945<br>p<0.0001	0.970<br>p<0.0001	1						
H_Tail_G1	0.930<br>p<0.0001	0.992<br>p<0.0001	0.921<br>p<0.0001	0.956<br>p<0.0001	0.975<br>p<0.0001	0.931<br>p<0.0001	0.687<br>p=0.028	0.883<br>p=0.001	0.899<br>p<0.0001	0.957<br>p<0.0001	0.966<br>p<0.0001	0.969<br>p<0.0001	0.970<br>p<0.0001	0.955<br>p<0.0001	0.968<br>p<0.0001	0.988<br>p<0.0001	1					

Supplementary material S4. Correlation between physiological and morphometric parameters on dogs of brachycephalic biotype grade 2 BOAS subjected to mild exercise tests.

	HR_G2	RR_G2	SBP_G2	DBP_G2	MAP_G2	SPO2_G2	Tcore_G2	L_muzzle G2	L_craneal G2	W_eyes G2	L_neck G2	C_neck G2	C_chest G2	W_chest G2	L_body G2	H_wither G2	H_tail G2	RFL G2	LFL G2	RHL G2	LHL G2	E_Facial G2
HR_G2	1																					
RR_G2	0.894 p<0.0001	1																				
SBP_G2	0.946 p<0.0001	0.952 p<0.0001	1																			
DBP_G2	0.944 p<0.0001	0.974 p<0.0001	0.971 p<0.0001	1																		
MAP_G2	0.938 p<0.0001	0.967 p<0.0001	0.973 p<0.0001	0.996 p<0.0001	1																	
SPO2_G2	0.887 p<0.0001	0.709 p<0.0001	0.784 p<0.0001	0.772 p<0.0001	0.773 p<0.0001	1																
Tcore_G2	0.991 p<0.0001	0.871 p<0.0001	0.938 p<0.0001	0.927 p<0.0001	0.924 p<0.0001	0.913 p<0.0001	1															
L_muzzle_G2	0.917 p<0.0001	0.858 p<0.0001	0.781 p<0.0001	0.859 p<0.0001	0.818 p<0.0001	0.664 p=0.003	0.841 p<0.0001	1														
L_craneal_G2	0.946 p<0.0001	0.915 p<0.0001	0.839 p<0.0001	0.909 p<0.0001	0.888 p<0.0001	0.739 p<0.0001	0.881 p<0.0001	0.977 p<0.0001	1													
W_eyes_G2	0.985 p<0.0001	0.956 p<0.0001	0.953 p<0.0001	0.977 p<0.0001	0.950 p<0.0001	0.858 p<0.0001	0.969 p<0.0001	0.89 p<0.0001	0.926 p<0.0001	1												
L_neck_G2	0.964 p<0.0001	0.901 p<0.0001	0.851 p<0.0001	0.912 p<0.0001	0.874 p<0.0001	0.749 p<0.0001	0.904 p<0.0001	0.974 p<0.0001	0.978 p<0.0001	0.942 p<0.0001	1											
C_neck_G2	0.874 p<0.0001	0.862 p<0.0001	0.765 p<0.0001	0.843 p<0.0001	0.827 p<0.0001	0.673 p=0.002	0.817 p<0.0001	0.961 p<0.0001	0.971 p<0.0001	0.863 p<0.0001	0.928 p<0.0001	1										
C_chest_G2	0.776 p<0.0001	0.782 p<0.0001	0.692 p=0.001	0.777 p<0.0001	0.764 p<0.0001	0.615 p=0.007	0.734 p=0.001	0.863 p<0.0001	0.872 p<0.0001	0.793 p<0.0001	0.829 p<0.0001	0.934 p<0.0001	1									
W_chest_G2	0.941 p<0.0001	0.916 p<0.0001	0.860 p<0.0001	0.915 p<0.0001	0.891 p<0.0001	0.760 p<0.0001	0.894 p<0.0001	0.959 p<0.0001	0.972 p<0.0001	0.937 p<0.0001	0.964 p<0.0001	0.953 p<0.0001	0.920 p<0.0001	1								
L_Body_G2	0.675 p=0.002	0.702 p=0.001	0.608 p=0.007	0.694 p<0.0001	0.684 p=0.002	0.541 p=0..021	0.650 p=0.004	0.784 p<0.0001	p<0.0001	0.707 p=0.001	0.730 p=0.001	0.876 p<0.0001	0.980 p<0.0001	0.854 p<0.0001	1							
H_wither_G2	0.964 p<0.0001	0.953 p<0.0001	0.902 p<0.0001	0.960 p<0.0001	0.939 p<0.0001	0.807 p<0.0001	0.924 p<0.0001	0.947 p<0.0001	0.972 p<0.0001	0.959 p<0.0001	0.953 p<0.0001	0.933 p<0.0001	0.866 p<0.0001	0.975 p<0.0001	0.797 p<0.0001	1						
H_Tail_G2	0.844 p<0.0001	0.921 p<0.0001	0.911 p<0.0001	0.913 p<0.0001	0.951 p<0.0001	0.970 p<0.0001	0.879 p<0.0001	0.720 p=0.001	0.793 p<0.0001	0.846 p<0.0001	0.780 p<0.0001	0.750 p<0.0001	0.700 p=0.001	0.805 p<0.0001	0.638 p=0.004	0.844 p<0.0001	1					
RFL_G2	0.900 p<0.0001	0.895 p<0.0001	0.807 p<0.0001	0.885 p<0.0001	0.870 p<0.0001	0.717 p=0.001	0.846 p<0.0001	0.946 p<0.0001	0.976 p<0.0001	0.903 p<0.0001	0.938 p<0.0001	0.986 p<0.0001	0.932 p<0.0001	0.960 p<0.0001	0.864 p<0.0001	0.946 p<0.0001	0.785 p<0.0001	1				
LFL_G2	0.892 p<0.0001	0.873 p<0.0001	0.787 p<0.0001	0.867 p<0.0001	0.850 p<0.0001	0.695 p=0.001	0.832 p<0.0001	0.965 p<0.0001	0.975 p<0.0001	0.883 p<0.0001	0.936 p<0.0001	0.993 p<0.0001	0.940 p<0.0001	0.966 p<0.0001	0.887 p<0.0001	0.954 p<0.0001	0.773 p<0.0001	0.981 p=0.000	1			
RHL _G2	0.962 p<0.0001	0.970 p<0.0001	0.930 p<0.0001	0.964 p<0.0001	0.951 p<0.0001	0.846 p<0.0001	0.956 p<0.0001	0.923 p<0.0001	0.960 p<0.0001	0.969 p<0.0001	0.940 p<0.0001	0.936 p<0.0001	0.862 p<0.0001	0.960 p<0.0001	0.790 p<0.0001	0.979 p<0.0001	0.865 p<0.0001	0.953 p=0.000	0.944 p=0.000	1		

Supplementary material S5. Correlation between physiological and morphometric parameters on dogs of brachycephalic biotype grade 3 BOAS subjected to mild exercise tests.

	HR_G3	RR_ G3	SBP_ G3	DBP_ G3	MAP_G3	SPO2_ G3	Tcore_ G3	L_muzzle G3	L_craneal G3	W_eyes G3	L_neck G3	C_neck G3	C_chest G3	W_chest G3	L_Body G3	H_wither G3	H_tail G3	RFL G3	LFL G3	RHL G3	LHL G3	E_Facial G3
HR_G3	1																					
RR_G3	0.929 p<0.0001	1																				
SBP_G3	0.948 p<0.0001	0.957 p<0.0001	1																			
DBP_G3	0.941 p<0.0001	0.943 p<0.0001	0.962 p<0.0001	1																		
MAP_G3	0.946 p<0.0001	0.940 p<0.0001	0.965 p<0.0001	0.997 p<0.0001	1																	
SPO2_G3	0.946 p<0.0001	0.976 p<0.0001	0.961 p<0.0001	0.973 p<0.0001	0.977 p<0.0001	1																
Tcore_G3	0.954 p<0.0001	0.977 p<0.0001	0.929 p<0.0001	0.964 p<0.0001	0.963 p<0.0001	0.972 p<0.0001	1															
L_muzzle_G3	0.744 p=0.005	0.981 p<0.0001	0.922 p<0.0001	0.909 p<0.0001	0.826 p=0.001	0.891 p<0.0001	0.879 p<0.0001	1														
L_craneal_G3	0.593 p=0.042	0.912 p<0.0001	0.819 p=0.001	0.806 p=0.002	0.714 p=0.009	0.756 p=0.004	0.768 p=0.004	0.949 p<0.0001	1													
W_eyes_G3	0.890 p<0.0001	0.872 p<0.0001	0.905 p<0.0001	0.959 p<0.0001	0.959 p<0.0001	0.865 p<0.0001	0.931 p<0.0001	0.904 p<0.0001	0.848 p<0.0001	1												
L_neck_G3	0.735 p=0.0006	0.958 p<0.0001	0.948 p<0.0001	0.875 p<0.0001	0.822 p=0.001	0.908 p<0.0001	0.847 p=0.001	0.974 p<0.0001	0.946 p<0.0001	0.911 p<0.0001	1											
C_neck_G3	0.670 p=0.017	0.929 p<0.0001	0.874 p<0.0001	0.878 p<0.0001	0.782 p=0.003	0.820 p=0.001	0.815 p=0.001	0.961 p<0.0001	0.956 p<0.0001	0.893 p<0.0001	0.963 p<0.0001	1										
C_chest_G3	0.640 p=0.025	0.860 p<0.0001	0.855 p<0.0001	0.808 p=0.001	0.770 p=0.003	0.817 p=0.001	0.751 p=0.005	0.908 p<0.0001	0.897 p<0.0001	0.894 p<0.0001	0.934 p<0.0001	0.926 p<0.0001	1									
W_chest_G3	0.652 p=0.022	0.915 p<0.0001	0.860 p<0.0001	0.847 p=0.001	0.779 p=0.003	0.811 p=0.001	0.797 p=0.002	0.956 p<0.0001	0.961 p<0.0001	0.902 p<0.0001	0.961 p<0.0001	0.972 p<0.0001	0.977 p<0.0001	1								
L_Body_G3	0.935 p<0.0001	0.795 p=0.002	0.857 p<0.0001	0.952 p<0.0001	0.989 p<0.0001	0.847 p=0.001	0.951 p<0.0001	0.784 p=0.003	0.685 p=0.014	0.945 p<0.0001	0.790 p=0.002	0.753 p=0.005	0.731 p=0.007	0.740 p=0.006	1							
H_wither_G3	0.623 p=0.031	0.968 p<0.0001	0.882 p<0.0001	0.831 p=0.001	0.731 p=0.007	0.866 p<0.0001	0.808 p=0.001	0.976 p<0.0001	0.952 p<0.0001	0.826 p=0.001	0.967 p<0.0001	0.954 p<0.0001	0.900 p<0.0001	0.951 p<0.0001	0.688 p=0.013	1						
H_Tail_G3	0.666 p=0.018	0.964 p<0.0001	0.897 p<0.0001	0.865 p<0.0001	0.781 p=0.003	0.867 p<0.0001	0.835 p=0.001	0.983 p<0.0001	0.972 p<0.0001	0.881 p<0.0001	0.983 p<0.0001	0.978 p<0.0001	0.933 p<0.0001	0.976 p<0.0001	0.746 p=0.005	0.991 p<0.0001	1					
RFL_G3	0.647 p=0.023	0.838 p=0.001	0.839 p=0.001	0.791 p=0.002	0.749 p=0.005	0.745 p=0.005	0.727 p=0.007	0.886 p<0.0001	0.929 p<0.0001	0.898 p<0.0001	0.931 p<0.0001	0.932 p<0.0001	0.957 p<0.0001	0.961 p<0.0001	0.737 p=0.006	0.871 p<0.0001	0.920 p<0.0001	1				
LFL_G3	0.656 p=0.021	0.876 p<0.0001	0.854 p=0.000	0.821 p=0.001	0.770 p=0.003	0.866 p=0.004	0.762 p=0.004	0.910 p<0.0001	0.942 p<0.0001	0.904 p<0.0001	0.945 p<0.0001	0.952 p<0.0001	0.958 p<0.0001	0.976 p<0.0001	0.756 p=0.004	0.900 p<0.0001	0.943 p<0.0001	0.992 p<0.0001	1			
RHL _G3	0.632 p=0.028	0.778 p=0.003	0.784 p=0.003	0.772 p=0.003	0.742 p=0.006	0.699 p=0.011	0.697 p=0.012	0.833 p=0.001	0.859 p<0.0001	0.887 p<0.0001	0.870 p<0.0001	0.887 p<0.0001	0.960 p<0.0001	0.940 p<0.0001	0.731 p=0.007	0.807 p=0.001	0.865 p<0.0001	0.977 p<0.0001	0.971 p<0.0001	1		
LHL_G3	0.620 p=0.031	0.735 p=0.006	0.730 p=0.007	0.751 p=0.005	0.716 p=0.009	0.643 p=0.024	0.676 p=0.016	0.808 p=0.001	0.846 p=0.001	0.872 p<0.0001	0.837 p=0.001	0.869 p<0.0001	0.937 p<0.0001	0.923 p<0.0001	0.704 p=0.011	0.776 p=0.003	0.838 p=0.001	0.963 p<0.0001	0.952 p<0.0001	0.992 p<0.0001	1	
Ft_nasal_G3	0.910 p=0.004	0.951 p=0.001	0.975 p<0.0001	0.832 p=0.020	0.871 p=0.011	0.918 p=0.003	0.827 p=0.022	0.956 p=0.001	0.910 p=0.001	0.913 p=0.004	0.946 p=0.001	0.966 p<0.0001	0.831 p=0.002	0.893 p=0.007	0.864 p=0.012	0.745 p=0.055	0.866 p=0.012	0.980 p=0.000	0.976 p<0.0001	0.998 p<0.0001	0.950 p=0.001	1
